# A Comprehensive Study on Nanoparticle Drug Delivery to the Brain: Application of Machine Learning Techniques

**DOI:** 10.1021/acs.molpharmaceut.3c00880

**Published:** 2023-12-07

**Authors:** Amal Yousfan, Mhd Jawad Al Rahwanji, Abdulsamie Hanano, Hisham Al-Obaidi

**Affiliations:** † The School of Pharmacy, University of Reading, Reading RG6 6AD, U.K.; ‡ Department of Pharmaceutics and Pharmaceutical Technology, Pharmacy College, Al Andalus University for Medical Sciences, Tartus, AL Kadmous 00000, Syria; § Department of Computer Science, Saarland University, Saarbrücken, Saarbrücken 66123, Germany; ∥ Department of Molecular Biology and Biotechnology, Atomic Energy Commission of Syria (AECS), P.O. Box 6091, Damascus 00000, Syria

**Keywords:** nanoparticles, intranasal drug delivery, brain, AUC, prediction, linear regression, linear mixed-effects

## Abstract

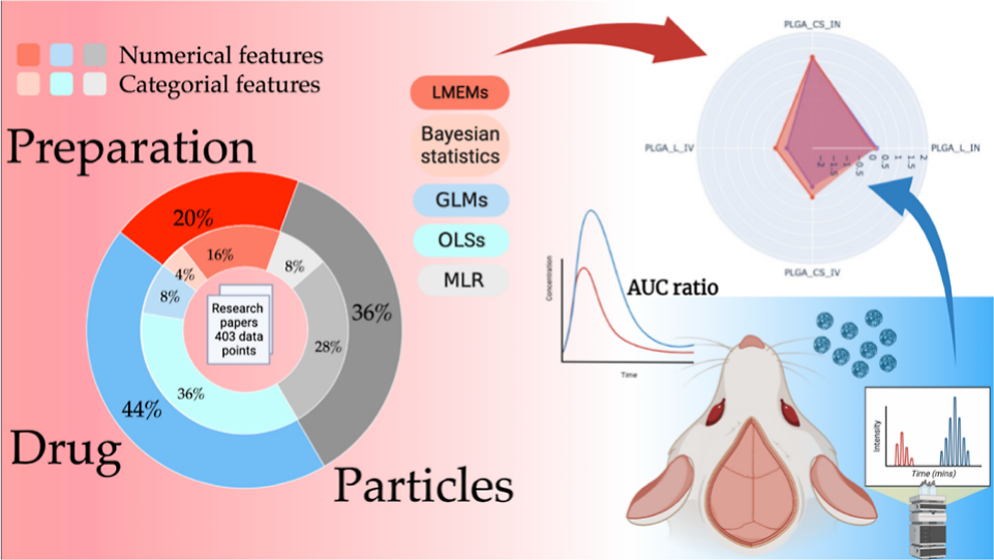

The delivery of drugs to specific target tissues and cells in the brain poses a significant challenge in brain therapeutics, primarily due to limited understanding of how nanoparticle (NP) properties influence drug biodistribution and off-target organ accumulation. This study addresses the limitations of previous research by using various predictive models based on collection of large data sets of 403 data points incorporating both numerical and categorical features. Machine learning techniques and comprehensive literature data analysis were used to develop models for predicting NP delivery to the brain. Furthermore, the physicochemical properties of loaded drugs and NPs were analyzed through a systematic analysis of pharmacodynamic parameters such as plasma area under the curve. The analysis employed various linear models, with a particular emphasis on linear mixed-effect models (LMEMs) that demonstrated exceptional accuracy. The model was validated via the preparation and administration of two distinct NP formulations via the intranasal and intravenous routes. Among the various modeling approaches, LMEMs exhibited superior performance in capturing underlying patterns. Factors such as the release rate and molecular weight had a negative impact on brain targeting. The model also suggests a slightly positive impact on brain targeting when the drug is a P-glycoprotein substrate.

## 1 Introduction

The treatment of diseases such as Alzheimer’s, Parkinson’s, multiple sclerosis, epilepsy, and brain tumors is significantly hindered by the limited permeability of the blood–brain barrier (BBB). This barrier poses a major challenge, as less than 1% of administered drugs can effectively reach the central nervous system (CNS), where these diseases manifest. The resistant nature of the BBB restricts access of drugs to the CNS, impeding the treatment of CNS disorders. Understanding and overcoming the limitations of BBB permeability is crucial for advancing therapeutic interventions in these conditions.^1,2^

The structure and function of the BBB play a crucial role in regulating the transport of molecules to the CNS.^3^ Molecules can cross the BBB through various mechanisms, categorized as passive or active transport. Whether facilitated by cells or proteins, the receiving cell controls this molecular movement.^4,5^ While some molecules possess specific structural features for specialized transport, many rely on passive diffusion as the primary mechanism of transport.^6^ However, the molecular size adds complexity to drug transport, as molecules with a molecular weight above 400–500 Da are unlikely to passively diffuse across the BBB.^1^ The lipophilicity of a molecule directly influences its permeability, as the lipid solubility of a drug determines its ability to traverse the BBB.^3,5^ Several studies have investigated the relationship between the lipophilic nature of molecules and BBB permeability. Optimal permeation across the BBB has been suggested within a partitioning coefficient (log *P*) range of 1.5–2.7.^7−9^ Likewise, the log AUC brain/AUC plasma ratio has been identified as a reliable predictor of brain permeability.^4^

Considering these challenges, the intranasal (IN) route of drug delivery has shown promise in bypassing the BBB and directly delivering drugs to the brain.^10,11^ This approach reduces side effects and systemic exposure by delivering the drug directly through the trigeminal and olfactory pathways, requiring lower doses to achieve therapeutic effects.^12^ Moreover, IN drug delivery offers practical advantages, such as ease of administration, leading to increased patient compliance.^13^ However, IN drug delivery has limitations, including a high rate of mucociliary clearance and low permeability. To overcome these limitations, various approaches have been employed, such as the use of chemical penetration enhancers or formulations with mucoadhesive properties.^10^ Effective drug delivery systems must evade clearance by the immune and reticuloendothelial systems, penetrate the BBB, and reach specific cells within the complex tissue microenvironment.^11,14^ Among the different strategies, nanoparticles (NPs) have demonstrated the ability to permeate the BBB and facilitate deep penetration of drugs into brain tissues.^1,15^

Various types of NPs with distinct physicochemical properties have been investigated to improve drug delivery to the brain.^4,14,16^ Despite the common practice of evaluating the efficacy of nasal drug delivery using cell culture experiments, in vitro uptake may not accurately reflect in vivo conditions.^17^ Additionally, many studies lack comprehensive characterization of the prepared NPs, and there is often ambiguity regarding the normalization of administered doses based on body weight in in vivo studies. Consequently, the experimental characterization falls short in providing sufficient support for the generation and decision-making process in pharmaceutical development.^18^

Machine learning has the potential to revolutionize drug delivery by leveraging extensive experimental data to build predictive models. However, achieving optimal accuracy requires selecting a high-performing model that considers various data types, behavioral measures, and interdependencies among features.^18^ In the field of nanotechnology, machine learning has been successful in predicting various properties of nanomaterials and their behavior in biological environments. The absence of interpretability in these models presents a significant constraint to making informed decisions pertaining to the design and optimization of nanomaterials. Therefore, it is crucial to develop models that offer interpretability to ensure that the design and optimization of nanomaterials are carried out seamlessly. Additionally, machine learning models heavily rely on the quality and quantity of training data, which can lead to suboptimal performance when faced with data significantly different from those in the training set. Therefore, careful consideration must be given to selecting training data and validating models to ensure both accuracy and generalizability.^19−21^

There have been studies that developed predictive models to optimize drug delivery efficiency. Baghaei et al. employed artificial neural networks to predict the NP size and its correlation with the initial burst rate, considering factors such as the molecular weight of polylactic-*co*-glycolic acid (PLGA), solution concentration, and molecular weight of poly(vinyl alcohol).^18−21^ In a different study, Gao et al. demonstrated that combining chemical features and clinical phenotypes was more effective in predicting BBB permeability compared to using chemical features alone.^6,22^ Saini and Srivastava identified important physicochemical properties for predicting the biological activities of nanomaterials, including surface charge, corona, aggregation, and solubility.^23^ Another study by Shafaei and Khayati focused on using machine learning to predict the size of NPs, considering parameters such as reaction time, reagent concentration, Au salt-to-stabilizer concentration ratio, intensity, wavelength, and focusing conditions, primarily in the context of in vitro responses.^24^

Our study aims to determine the factors that affect the targeting of the brain, such as the properties of drugs, the method of preparation, and the properties of the prepared NPs. We used various linear models in our analysis as they provide better interpretability than other machine learning approaches. Linear mixed-effect models (LMEMs) are particularly useful in capturing both within-subject and between-subject effects, allowing for the incorporation of correlations between measurements obtained from the same individual. These models have shown superiority over traditional linear regression models by providing greater flexibility and accommodating a wider range of data structures.^25^

To the best of our knowledge, there are no comprehensive studies that have utilized a large data set and incorporated a combination of numerical and categorical features to predict the ratio between AUC brain and AUC plasma for brain targeting purposes. The objective of this research is to create an accurate model that predicts drug biodistribution in the brain and systemic circulation. This model takes into consideration the relevant physicochemical characteristics of the drug and nanocarriers. By leveraging this information, we aim to develop a reliable predictive tool specifically designed for brain targeting that would significantly reduce experimental costs and be instrumental in the design of optimal nanocarriers.

## 2 Materials and Methods

### 2.1 Materials

Phenytoin (PHT) and 5,5-diphenylhydantoin (batch #PB/10/14) were purchased from JPN PHARMA, India. Low-molecular-weight chitosan (batch #STBF8219 V), acetic acid, triacetin (batch #MKBC5147), and dialysis sacks (MWCO 12,000 Da) (batch #SLBQ 4638 V) were purchased from Sigma-Aldrich Co., Germany. Lecithin (phosphatidylcholine 51.9% and phosphatidylethanolamine 12.5%) (batch no. 20617HHFEA) was supplied by Cargill Co., Germany. Acetone and ethanol were purchased from Eurolab, UK. Methanol and chloroform were purchased from Merck, Germany. Poloxamer 188 was purchased from AppliChem, Germany. Xylazine (batch no. 358518) was purchased from Interchemie, Estonia. Ketamine hydrochloride (batch no. 50461) was purchased from Rotexmedica, Germany.

### 2.2 Data Collection

The study utilized data obtained from the PubMed and Science Direct databases. The inclusion criteria resulted in 237 research papers related to the preparation of NPs and involved administration via the nasal route compared to other reference routes, typically the intravenous (IV) route. The papers were meticulously selected, adhering to specific criteria to guarantee the inclusion of a comprehensive analysis of the physicochemical properties of NPs. These properties encompassed size, surface charge, encapsulation efficiency (EE) percentage, drug localization (core/shell), shape, and surface ligands or modifications. Additionally, the required papers provide detailed insights into the release studies. Furthermore, it was essential for them to report crucial data, including exposure time, drug accumulation, and in vivo evaluations, incorporating parameters such as the area under the curve (AUC), Tmax, and Cmax in both the plasma and brain. Inclusion criteria for eligible studies required the primary indicator of brain targeting to be AUC_brain_/AUC_plasma_ (*Y*) as well as Cmax_brain_/Cmax_plasma_ and Tmax_brain_/Tmax_plasma_. The features included both discrete and continuous variables. The resulting design matrix had 403 rows and 24 columns.

### 2.3 Exploratory Data Analysis

The aim of the current study was to examine the relationship between the different characteristics of NPs and their ability to target the brain. To assess this relationship, Pearson’s correlation coefficient was employed, indicating no significant correlation among the quantitative predictors or between drug targeting efficiency (DTE %) and direct transport percentage (DTP %) with the studied features (Supporting Information 1). The findings were further supported by conducting principal component analysis (Supporting Information 2).

**Figure 1:**
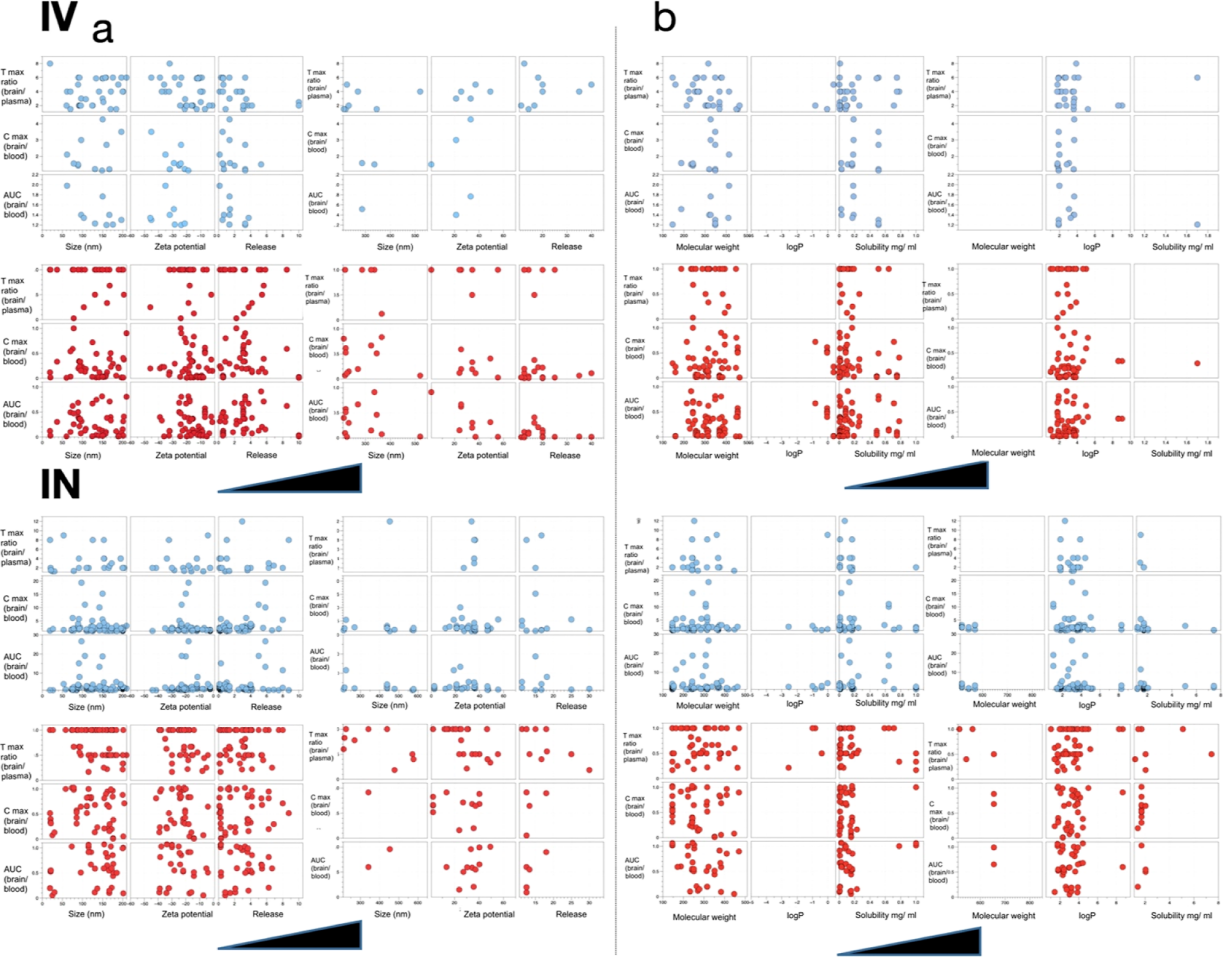
Network chart showing data grouped according to the target variable while taking the ratio = 1 as a cutoff. Red for <1 and blue >1. With respect to the cutoff for each studied feature, the resulting network chart shows the effect of administration route on AUC, Tmax, and Cmax ratios.

To address this issue, a comprehensive categorization of features was performed based on pharmaceutical considerations, and the results were presented visually. The categorization criteria included specific cutoff values: 500 Da for molecular weight, 0 for log *P*, 0.2 mg/mL for drug solubility, and 20% for release ratio %. Additionally, a particle size cutoff of 200 nm was chosen, as it represents the theoretical limit for particles to cross cellular membranes.^49^ The NPs were further classified based on their surface charge, categorized as either neutral or slightly negative, or positive, which can affect their mucoadhesive properties. To facilitate comparison among different drugs with varying doses, ratios such as the brain to plasma AUC ratio, Cmax, and Tmax were utilized. These categorizations and ratios are visually depicted in Figure 1 and 3.

To ensure the quality of the data, several steps were taken. These included the addition of missing values, removal of duplicate data and outliers, and elimination of collinear predictors. The final data set consisted of 133 observations with a total of 12 predictors, including 4 qualitative and 8 quantitative predictors (Appendix 1). For certain tests, such as LMEMs and Bayesian inference, an alternative version of the data set was utilized. This alternative data set comprised the same set of 12 features while retaining the complete set of 403 observations (Appendix 2).

### 2.4 Statistical Modeling Using Linear Regression-Based Models

In this study, various statistical models based on linear regression were used to analyze the data. Multiple linear regression or ordinary least-squares (OLS) is employed to predict dependent variables using multiple independent variables. The coefficients obtained from linear regression represent the linear contribution of each predictor to predicting the response variable. The goodness of fit of the models was assessed using the coefficient of determination (*R*^2^). To address nonlinearity and correct response distribution, different transformations were applied to both the predictors and the response variable. These transformations included reciprocal, logarithmic, and Boxcox transformations of the response variable. As for predictors, polynomial transformations were used for continuous variables, while target and binary encoding techniques were applied to categorical and binary variables, respectively.

Generalized linear models (GLMs) were also used, which allow various residual distribution assumptions and nonidentity links. In some cases, only significant predictors were included in the regression models, while in other cases, the entire set of predictors was used. The complete data set, consisting of 403 data points, was analyzed using LMEMs with random effects for each subject, taking into account the variability among the test animals (rats and mice). Bayesian data analysis was conducted on the complete data set as well. The aim of LMEMs and Bayesian statistics was to develop predictive models for the true distributions of the AUC. Statistical analysis was performed using software such as GraphPad Prism version 9.0 and Python 3.9 libraries, including SciPy,^50^ category encoders, statsmodels,^51^ Bambi,^52^ ArviZ, NumPy, and pandas. Data visualization was carried out using seaborn,^53^ and matplotlib.

### 2.5 Preparation of Validation NPs

To validate the model’s predictive ability for brain targeting, two different formulations of NPs loaded with PHT, an antiepileptic drug, were prepared. The nanoprecepitation method, based on the work of Yousfan et al. with some modifications,^54^ was used to prepare the NPs. After extensive optimization (data not shown), a standardized protocol was established, and the specific amounts of ingredients were determined.

The first formulation, known as polylactic-*co*-glycolic acid chitosan NPs (PLGA CS-NPs), was prepared as follows: initially, 2.5 mg of PLGA was dissolved in 5 mL of organic phase comprising a mixture of ethanol and acetone (40:60% v/v). Subsequently, 1.2 mg of PHT was dissolved in 0.2 mL of triacetin and added to the organic phase. Meanwhile, 1.25 mg of chitosan was suspended in 10 mL of deionized water, and the pH was adjusted using acetic acid to achieve a chitosan/acetic acid solution with a ratio of 1:1.75 (w/v). To stabilize the solution, a nonionic surfactant called poloxamer 188 was added to the chitosan solution at a concentration of 0.2%. The organic phase was then added dropwise into the aqueous phase while stirring at a speed of 600 rpm and at room temperature. The resulting suspension was evaporated under low pressure using a rotary evaporator at a vacuum of 168 mbar and a temperature of 70 °C. Subsequently, the NP suspension was subjected to centrifugation using Viva-spin 100 kDa MWCO centrifugal concentrators at 3214 g for 2 h at 20 °C to separate the NPs from soluble nonreactive components. Finally, the NPs were collected from the upper chamber of the viva-spin tubes. The second formulation, polylactic-*co*-glycolic acid lecithin NPs (PLGA L-NPs), followed a similar process with slight modifications. In this case, 2.5 mg of PLGA and 5 mg of lecithin were dissolved in the 5 mL organic phase consisting of ethanol and acetone (40:60% v/v). The rest of the procedure remained the same, including the addition of 1.2 mg of PHT dissolved in 0.2 mL of triacetin, the inclusion of 0.2% poloxamer 188 in the aqueous phase, and the subsequent steps.

These formulations were specifically used to evaluate the accuracy of the model’s predictions for brain targeting. The main goal was to assess the effectiveness of the NPs in delivering PHT to the brain and validate the reliability of the model in predicting these outcomes. To ensure an unbiased evaluation, experiments involving the preparation and evaluation of the NPs were conducted by one group, while the statistical tests and analysis were performed by another group of individuals. This approach prevented any exchange of information between the two groups and introduced a double-blinded evaluation process to minimize bias.

### 2.6 SEM Imaging and DLS

Scanning electron microscopy (SEM) imaging and dynamic light scattering (DLS) techniques were employed to analyze the characteristics of the prepared NPs. For SEM imaging, a VIGA II Xmu scanning electron microscope (TESCAN, Czechia) operating at an accelerating voltage of 20 kV was used. The morphology of the NPs was examined by using a secondary electron detector with a magnification of 3000 kV and a scan speed of 8.

DLS measurements were conducted to determine the average dynamic size, size distribution, and surface charge (zeta potential) of the NPs. A Malvern Zetasizer instrument (UK) was utilized for these measurements. Scattered light at an angle of 90° was collected for 2 min at a temperature of 25 °C. Each sample was subjected to 20 runs, with triplicate measurements within each run, and the average values were calculated.

### 2.7 EE Determination

To determine the EE of PHT in the prepared NPs, the amount of free PHT in the clear filtrate was analyzed by using high-performance thin-layer chromatography (HP-TLC). The TLC plate was developed using a chloroform/acetone solvent mixture (9:1, v/v), and the UV absorption of the tracks was measured at 217 nm using an HP-TLC scanner (CAMAG TLC scanner 3, Germany).^54^ The concentration of PHT was determined from a linear standard curve. The EE percentage was calculated by using the following formula: encapsulation efficiency (EE %) = (*W*_NP_/*W*_T_) × 100% where *W*_NP_ is the total amount of drug in the NPs and W_T_ the total quantity of drug added initially during preparation. Furthermore, the release of PHT from the NPs was investigated by suspending the NPs in 5 mL of deionized water and dialyzing them against 50 mL of deionized water using dialysis sacks with a cutoff at 12 kDa. The release medium was collected and replenished at specific time points. The collected samples were analyzed by using HP-TLC, and the data were plotted accordingly.

### 2.8 In Vivo Validation Experiments

In this study, we conducted an investigation of the brain delivery of PHT following the IN and IV administration of prepared NPs in healthy female Balb/c mice. The mice used in the study were aged 12–16 weeks, weighed between 20 and 30 g, and randomly divided into four groups to ensure reliable and representative results. Conscious mice were subjected to noninvasive IN administration following the protocol described by Hanson et al.^11^ Briefly, 15 μL of NP suspension was dropped in each open nostril of a conscious mouse, enabling the delivery of the NP suspension toward the roof of the nasal cavity. Fifteen healthy female mice BALB/c, aged 12–16 weeks, and weighing 20–30 g were randomly divided into five groups. NPs with an encapsulated dose of 7 mg of PHT for every 1 mL of NP suspension were administered through the nasal cavity (15 μL in each nostril) or were injected in 100 μL of PBS via a single tail vein (IV). At 5 min, 15 min, 1 h, 4 h, and 24 h after the administration, mice were ethically euthanized using IP terminal anesthesia containing 87.5 and 12.5 mg/kg ketamine and xylazine, respectively.

Blood samples were taken by open cardiac puncture, and the brain was then isolated and weighed. The concentration of PHT in biological tissues was measured using Sykam HPLC with a UV/vis detector, Germany. The analysis was conducted using methanol/water (55:45) as the mobile phase, C8 (4.6 250 mm; 5 mm) as the stationary phase, 40 °C, 1 mL min^–1^ flow rate, and the resolved peaks were measured at 210 nm wavelength.

## 3 Results and Discussion

### 3.1 Data Extraction and Identification of Critical Factors

**Figure 2:**
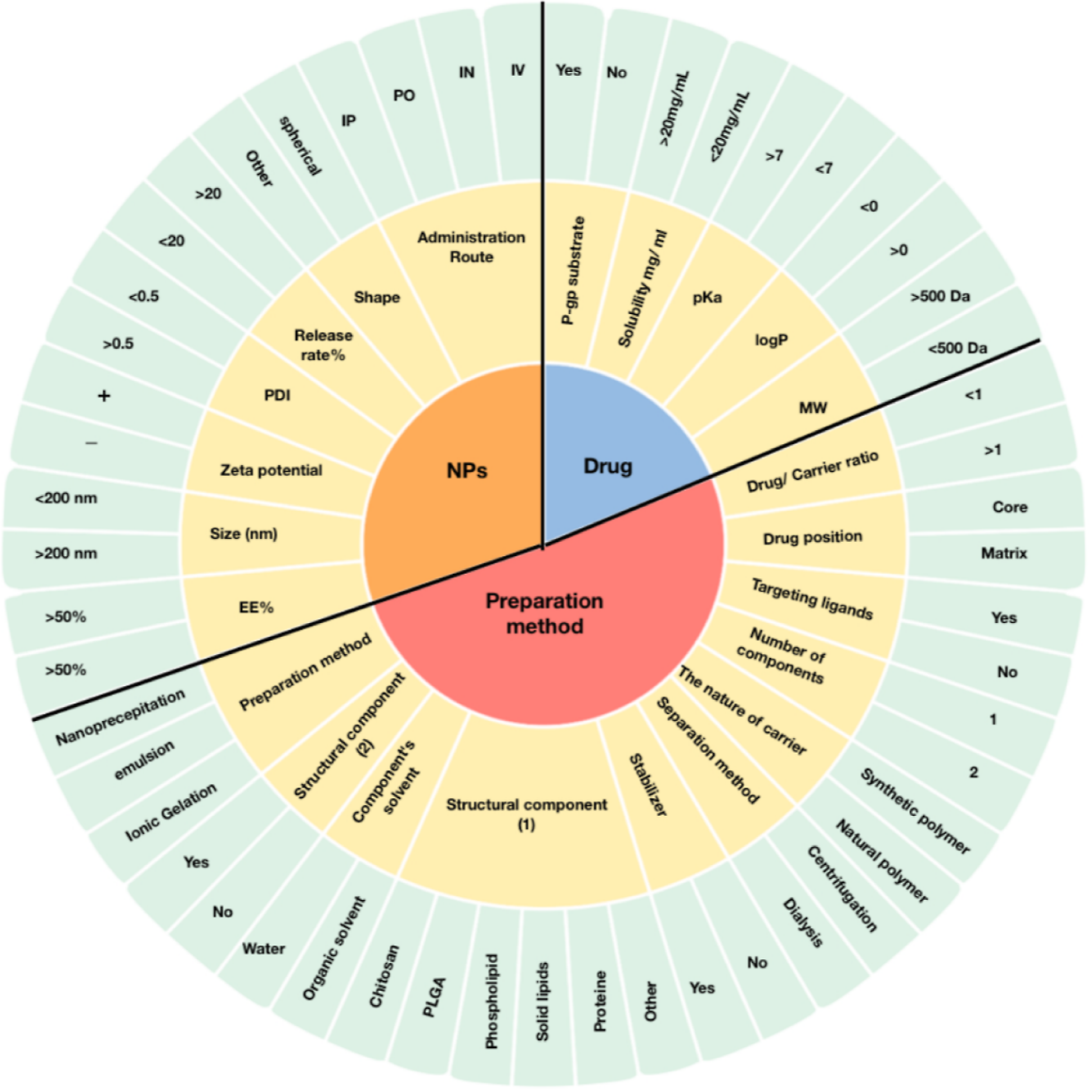
Krona chart showing the subgrouping of the data from the mined studies.

A meticulous selection process was undertaken to identify a set of 23 significant factors, previously established in studies, that are closely linked to the properties of drug encapsulation and the methods employed for the preparation of NPs.^26−28^ The relevant drug properties were obtained from the Drugbank database.^29^ The essential features included 5 variables related to the drug (i.e., molecular weight (*M*_w_), log *P*, p*K*_a_, solubility mg/mL, and whether the drug is P-gp substrate), 11 variables related to the preparation method (i.e., drug-carrier ratio, drug position, structural component i, structural component ii, number of components, carrier nature, preparation method, structural component’s solvent, stabilizer, separation method, targeting ligands), and 7 variables related to the physicochemical properties of prepared NPs (i.e., size (nm) zeta potential (μV), PDI, EE %, NPs’ shape, release rate %) as illustrated in Figure 2. The responses included AUC_brain_/AUC_plasma_ (*Y*) as the primary indicator of brain targeting, Cmax_brain_/Cmax_plasma_, and Tmax_brain_/Tmax_plasma_. The features consisted of discrete variables and continuous variables. The resulting design matrix consisted of 403 rows and 24 columns.

### 3.2 Statistical Analysis

#### 3.2.1 Multivariate Linear Regression

A multilinear regression analysis was performed to evaluate factors that affect brain targeting ability of NPs administered via the IV and the IN routes. The analysis included 8 dependent variables, transformations of the response variable (*Y*) and polynomial transformations of the predictor variables (*X*). These statistical tools were applied to achieve residual homoscedasticity, normality, and linearity, as presented in Tables 1 and 2. Predictors with coefficients having *p*-values below 0.05 were used for predictions.

**Table 1:** *R*^2^ Scores and Normality of Residuals for the Tested Transformations of *X* and *Y* that Resulted from MLR (IV Administration)

IV administration		*X*	*X*^2^	*X*^3^
*Y*	*R*^2^	0.7446	0.8298	0.8218
	normality of residuals	Anderson–Darling (A2*)	D’Agostino–Pearson omnibus (K2)	Anderson–Darling (A2*)
1/*Y*	*R*^2^	0.787	0.8807	0.8806
	normality of residuals	D’Agostino–Pearson omnibus (K2)	Kolmogorov–Smirnov (distance)	none
log(*Y*)	*R*^2^	0.8276	0.8753	0.8769
	normality of residuals	D’Agostino–Pearson omnibus (K2)	Kolmogorov–Smirnov (distance)	Kolmogorov–Smirnov (distance)

**Table 2:** *R*^2^ Scores and Normality of Residuals for the Tested Transforms of *X* and *Y* that Resulted from MLR (IN Administration)

IN administration		*X*	*X*^2^	*X*^3^
*Y*	*R*^2^	0.6895	0.7398	0.7801
	normality of residuals	D’Agostino–Pearson omnibus (K2)	D’Agostino–Pearson omnibus (K2)	none
1/Y	*R*^2^	0.5546	0.7122	0.6895
	normality of residuals	none	Kolmogorov–Smirnov (distance)	Kolmogorov–Smirnov (distance)
log(*Y*)	*R*^2^	0.6237	0.7320	0.7445
	normality of residuals	Kolmogorov–Smirnov (distance)	Kolmogorov–Smirnov (distance)	Shapiro–Wilk (W)

For IV administration, *R*^2^ values greater than 0.85 were obtained, specifically 0.8769 and 0.8753 for *X*^3^ and *X*^2^ with log *Y*, respectively (Table 1). The analysis identified zeta potential, drug/carrier ratio, and release rate % as potential predictors for the brain targeting ability of NPs following IV administration. These factors play a crucial role in the interaction of NPs with the in vivo environment and their ability to target the brain.^30,31^ Similarly, for IN administration, *R*^2^ values exceeding 0.7 were achieved, specifically 0.7445 and 0.7320 for *X*^3^ and *X*^2^ with log *Y*, respectively (Table 2). A variety of factors, including molecular weight, drug solubility, log *P*, size (nanometers), and zeta potential (μV), were found to influence the brain targeting pattern after IN administration. The measured properties of the prepared NPs and the coefficient estimates in Table 3 show a summary of correlations between these factors and the brain targeting response.

**Table 3:** Coefficient Estimates from the MLR Analysis across 4 Models: Two Models per Polynomial Transformation of *X* and Two Models per Administration Route

	*X*^3^ + log(*Y*) IN	*X*^2^ + log(*Y*) IN	*X*^3^ + log(*Y*) IV	*X*^2^ + log(*Y*) IV
intercept	–2.193	–0.128	–0.794	0.093
molecular weight	0.01119			
release				–0.329
molecular weight*solubility	–0.03215	–0.02784		
solubility*zeta potential	–0.04813	–0.0431		
solubility*release	0.5133	0.534		0.0325
size*zeta potential	0.0001244			
zeta potential*release	–0.001369	–0.0012	0.0325	0.0325
log(*P*)*size		0.0028		
size*release		0.000541		
releasê2				0.00624
releasê3			0.000242	

#### 3.2.2 Ordinary Least Squares

In this approach, there are two key differences compared to multivariate linear regression (MLR) analysis. First, categorical variables were target encoded, allowing for their inclusion in the analysis. This resulted in a design matrix with 12 predictors. Second, the administration routes were not separated. Additionally, various predictor transformations and interactions were explored, and response transformations, such as Boxcox and log, were applied to meet the assumptions of OLS. The models were evaluated using adjusted *R*^2^ and AIC.

**Figure 3:**
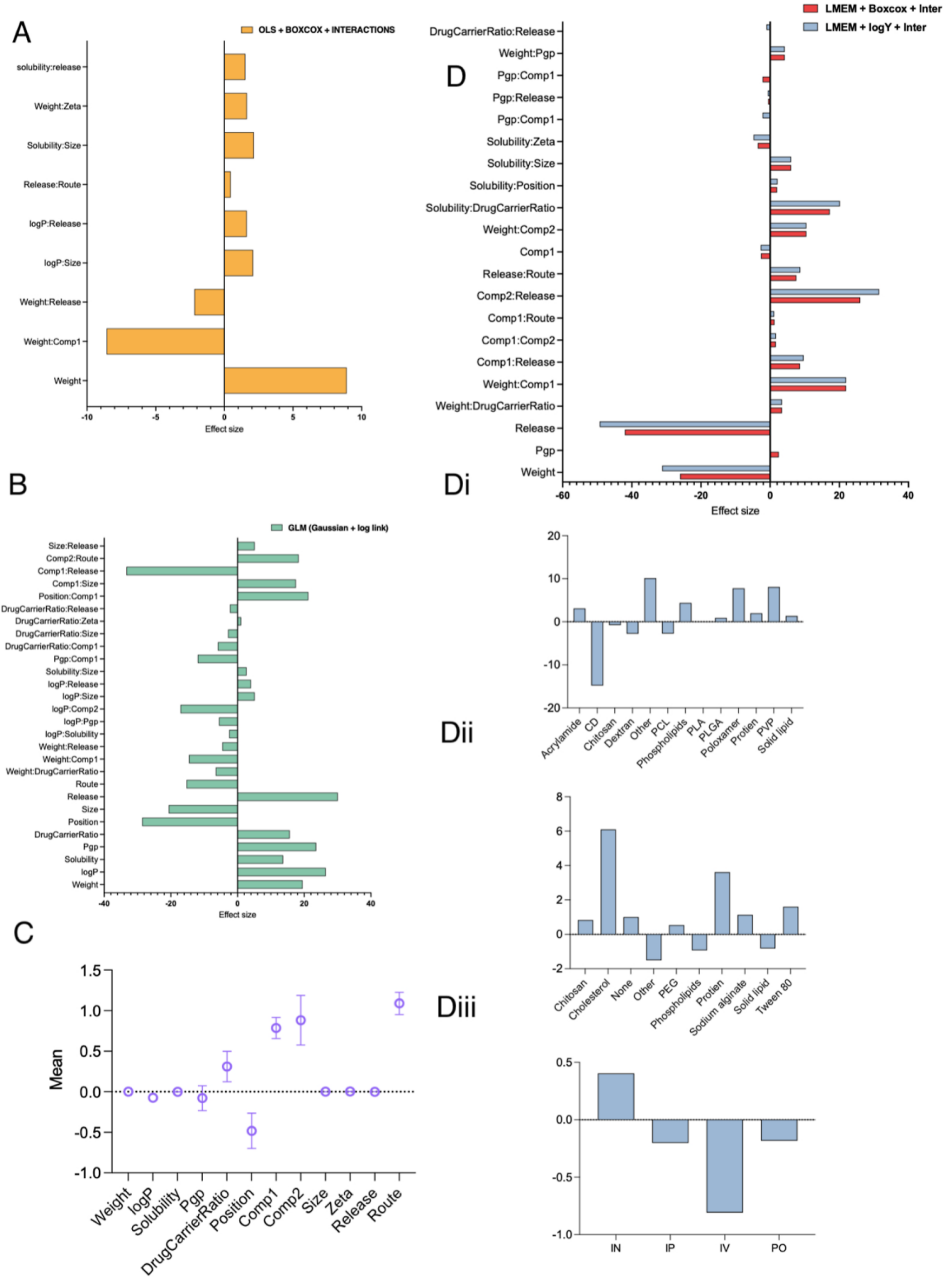
A,B,D) Coefficient estimates of the significant descriptors in different models. The values were normalized to assert the input space scale invariance. Di through Diii showing AUC means per type of comp 1, comp 2, and route. (C) Posterior distribution characteristic of the descriptor coefficients after Bayesian analysis.

Table 4 shows that several models achieved adjusted *R*^2^ scores of approximately 0.75 or higher along with low AIC scores and mean square error (MSE) values. These models included 2-way interactions, indicating that the predictive power of the predictors increases when they interact, especially considering that none of the predictors had a direct correlation with the response variable. Furthermore, the inclusion of target-encoded categorical variables resulted in highly adjusted *R*^2^ values. However, the combination of administration routes led to an improvement over the MLR model but increased the complexity of the model. The response variable displayed a positive linear relationship with the molecular weight of the encapsulated drug. In contrast, a notable negative linear relationship was observed for the weight/comp 1 interaction. This negative effect was inverted due to the negative mean of the target encoded comp1 variable. The combination of release and molecular weight had a negative impact on the response variable, as shown in Figure 3A.

**Table 4:** Results from the OLS Experiments and Fitted Models

model	*R*^2^	adj. *R*^2^	MSE	AIC	model dof	prob (Jarque–Bera)	conditional number	(Breusch–Pagan) *p*-value
OLS	0.419	0.361		363.3	12	1.13 × 10–12	8.75 × 103	0.0037
OLS + Boxcox *Y* transform	0.503	0.454		347.7	12	0.9	8.75 × 103	0.089
OLS + Boxcox +2-way interactions	0.896	0.745	0.339	272.0	78	0.335	7.08 × 107	0.17
OLS + 2-way interactions	0.849	0.631	0.472	315.9	78	2.12 × 10–6	7.08 × 107	0.91
OLS + log *Y* + 2-way interactions	0.913	0.788	0.469	315.1	78	0.172	7.08 × 107	0.133
OLS + log *Y* + 2-way interactions – *X* transformations	0.958	0.576	0.543	227.0	119	0.00	1.03 × 1016	1

#### 3.2.3 Generalized Linear Models

In this analysis, we used the same design matrix as in the OLS model, but this time we did not apply any response transformations. Instead, we tested different residual distribution assumptions and used the log-link in conjunction with Gaussian residual assumption. We included the encoded categorical variables and 2-way interactions, as before. In this case, we used the pseudo-*R*^2^ metric to evaluate the models. Table 5 shows that the γ distribution and the Gaussian distribution with a log-link achieved pseudo-*R*^2^ scores of 0.91 and 0.99, respectively. The GLM models outperformed the OLS models, as indicated by the improved AIC, *R*^2^, and MSE scores. This improvement can be attributed to assuming the correct distribution, as it allows the residuals to naturally follow the assumed distribution, rather than transforming non-normal residuals into normal ones. Regarding the log-link approach, it differs from the OLS model with a log *Y* transformation in that it takes the logarithm of the mean, while the latter takes the mean of the logarithms. Figure 3B illustrates the main positive effects observed for weight, log *P*, solubility, PgP, drug carrier ratio, and release. On the other hand, the size and position variables exhibited negative effects.

**Table 5:** Results from the GLM Experiments and Fitted Models

model	pseudo *R*^2^	MSE	AIC	deviance	model dof	(Breusch–Pagan) *p*-value
GLM (Tweedie)		0.1085	nan	12.55	78	0.97
GLM (γ)	0.910	0.1279	230.664	33.395	78	0.99
GLM (Gaussian + log link)	0.99	0.0796	239.739	10.588	78	0.98

#### 3.2.4 Linear Mixed Effects Models

In the analysis using LMEM, we employed a different design matrix that included all 403 observations. To account for repeated measures, a per-subject random intercept was added. Interactions were included based on their superior performance observed with GLM and OLS models. Additionally, a Boxcox response transformation was applied, and the conditional *R*^2^ value was used as a measure of regression fitness. Although the *R*^2^ scores in Table 6 decreased to approximately 0.75, it is important to consider that we are working with a larger data set that includes additional levels for categorical variables and covers a broader range of input values. Therefore, comparing the *R*^2^ scores of this model to those of the others is not appropriate. Through validation, it was observed that the LMEM outperformed the GLM. Overall, comparison between the Boxcox and log *Y* transformations provided inconclusive results in terms of fitting, but after validation, the Boxcox transformation was clearly superior. The LMEM models using Boxcox and log *Y* transformations exhibited nearly identical trends in terms of positive and negative linear coefficient estimates. An ANOVA conducted on comp1 revealed that cyclodextrins had a significantly different mean AUC (negative) compared to the other compounds, while poloxamer and PVP had positive means. Regarding comp2, cholesterol, protein, sodium alginate, polyethylene glycol (PEG), and tween 80 had positive mean AUC values, whereas phospholipids and solid lipid had negative mean AUC values. In terms of the administration route, only IN showed a positive mean AUC, as illustrated in Figure 3D.

**Table 6:** Results from the LMEM Experiments and Fitted Models

model	conditional *R*^2^	scale	(Lagrange multiplier) *p*-value	(normality test) *p*-value
LMEM – *X* tranfsormations	0.641	6.072	0.002	1.325
LMEM + Boxcox	0.649	0.891	0.0012	0.0414
LMEM + Boxcox + 2-way interactions	0.773	0.6192		0.0003
LMEM + log *Y* + 2-way interactions	0.767	0.731		4.9938

#### 3.2.5 Bayesian Inference

In the Bayesian inference analysis, we employed the entire data set with the same 12 predictors used previously, incorporating the necessary encodings. However, due to convergence issues, interactions were not included in this approach. While normality is not a requirement for Bayesian methods, a Boxcox transformation was applied to achieve the homoscedasticity of the residuals. Two models were fitted: one with a by-subject random intercept and one without. Table 7 displays the results, indicating that the model with the random intercept had a lower MSE. However, during the validation process, it became evident that this superiority did not hold when predicting unseen data. While the models performed well, they did not surpass the performance of the previous best models in terms of training or validation. Nevertheless, the advantage of Bayesian statistics lies in its ability to generate a posterior predictive distribution. If convergence is achieved, this distribution accurately represents the true distribution of the response variable. Consequently, we can confidently describe the probabilities associated with different outcomes and obtain a generative model for the target variable. A scatter plot, shown in Figure 3C, was generated to visualize the posterior distribution characteristics of the descriptor coefficients obtained from Bayesian analysis. The plot includes the mean value and standard deviation (mean ± standard deviation) of the coefficients.

**Table 7:** Results from the Bayesian Approach Experiments and Fitted Models

model	MSE
Bayesian statistics + Boxcox	1.7223
Bayesian statistics + Boxcox + by subject random intercept	0.5654

### 3.3 Model Validation Using Prepared NPs and In Vivo Assessment

In order to validate the models developed for predicting brain targeting, two types of NPs were prepared: PLGA L and PLGA CS NPs were loaded with PHT. These NPs were administered to mice via IN and IV routes. Detailed information about the drug properties, preparation method, and physicochemical characterization of the prepared NPs can be found in Table 8.

**Table 8:** Experimental Results Showing the Validation NP Properties

NPs	drug/carrier ratio	drug position	structural component (1)	structural component (2)	size (nm)	zeta potential	EE %	release ratio %	PDI
PLGA L-NPs	0.480	core	PLGA	phospholipids	170.630	–37.7	52.351	2.26	0.089
PLGA CS-NPs	0.480	matrix	PLGA	chitosan	453.100	33.4	56.412	2.95	0.338

**Figure 4:**
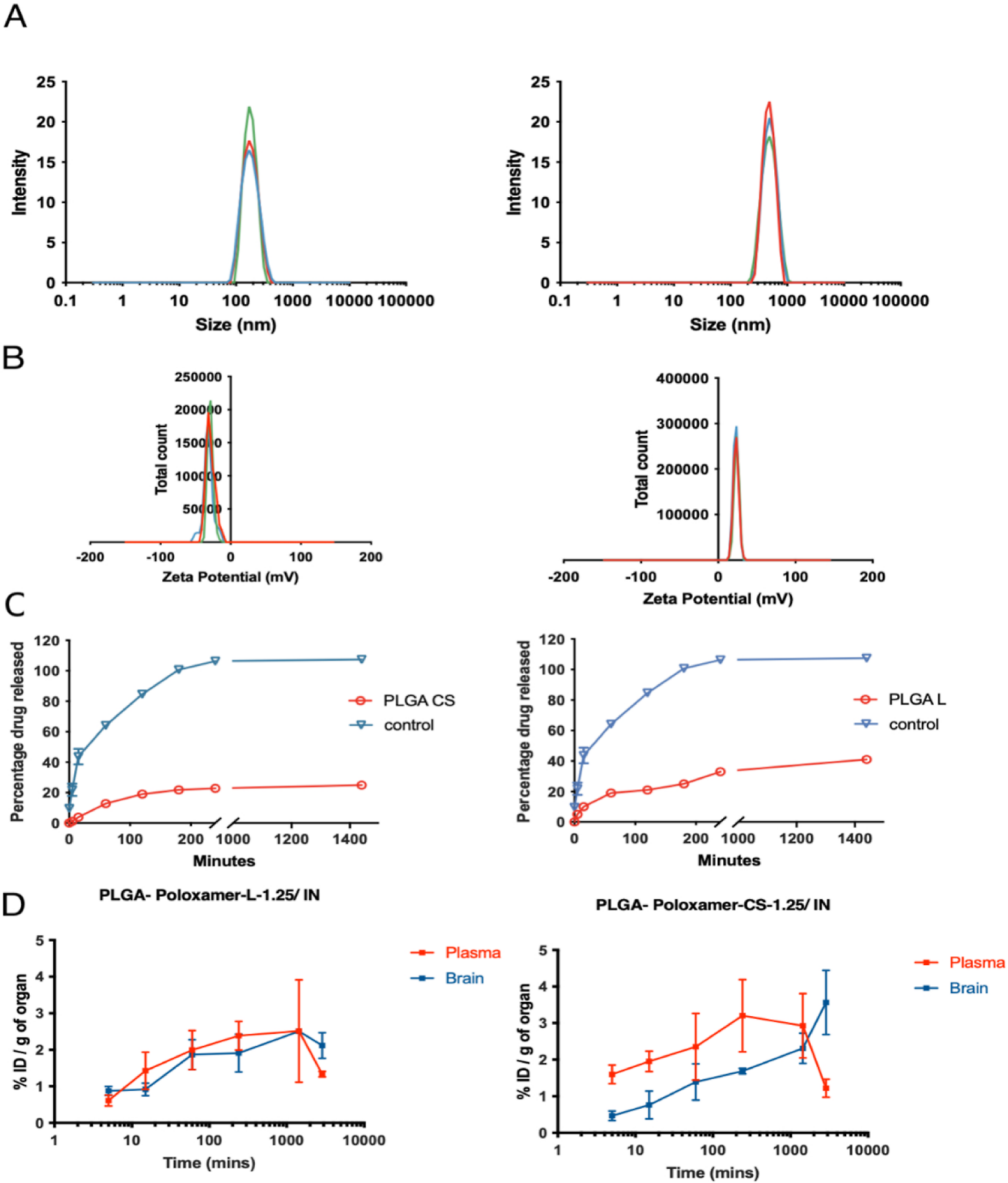
Particles size (A), zeta potential (B), and the release study (C) of PLGA L-NPs and PLGA CS-NPs using DLS and the dialysis method, respectively. The results are represented as mean percentage ± SD (*n* = 3 at least). (D) Uptake and concentration–time profile in brain and plasma following PLGA L-NPs and PLGA CS-NPs after IV and IN administration. The mean Cmax values in the brain after IN injection were found to be markedly greater than those obtained after IV administration for both PLGA L-NPs and PLGA CS-NPs. In the plasma and brain, PLGA L-NPs brought a faster onset of the PHT concentration. The brain AUC_0–∞_ after PLGA CS-NPs was higher than the PLGA L-NPs in plasma and brain. Brain exhibited the highest concentration of PHT after the IN administration of PLGA CS-NPs, while PLGA L-NPs exhibited the highest concentration of PHT after IV administration.

The analysis of drug distribution between the brain and plasma was performed to compare the experimental results to the predictions made by the models. Supporting Information 3 and Figure 4 present SEM photomicrographs and physicochemical properties of the prepared NPs, respectively, including size, zeta potential, release profile, and biodistribution after IN and IV administration. However, specific numerical values and data are not provided in this context.

**Figure 5:**
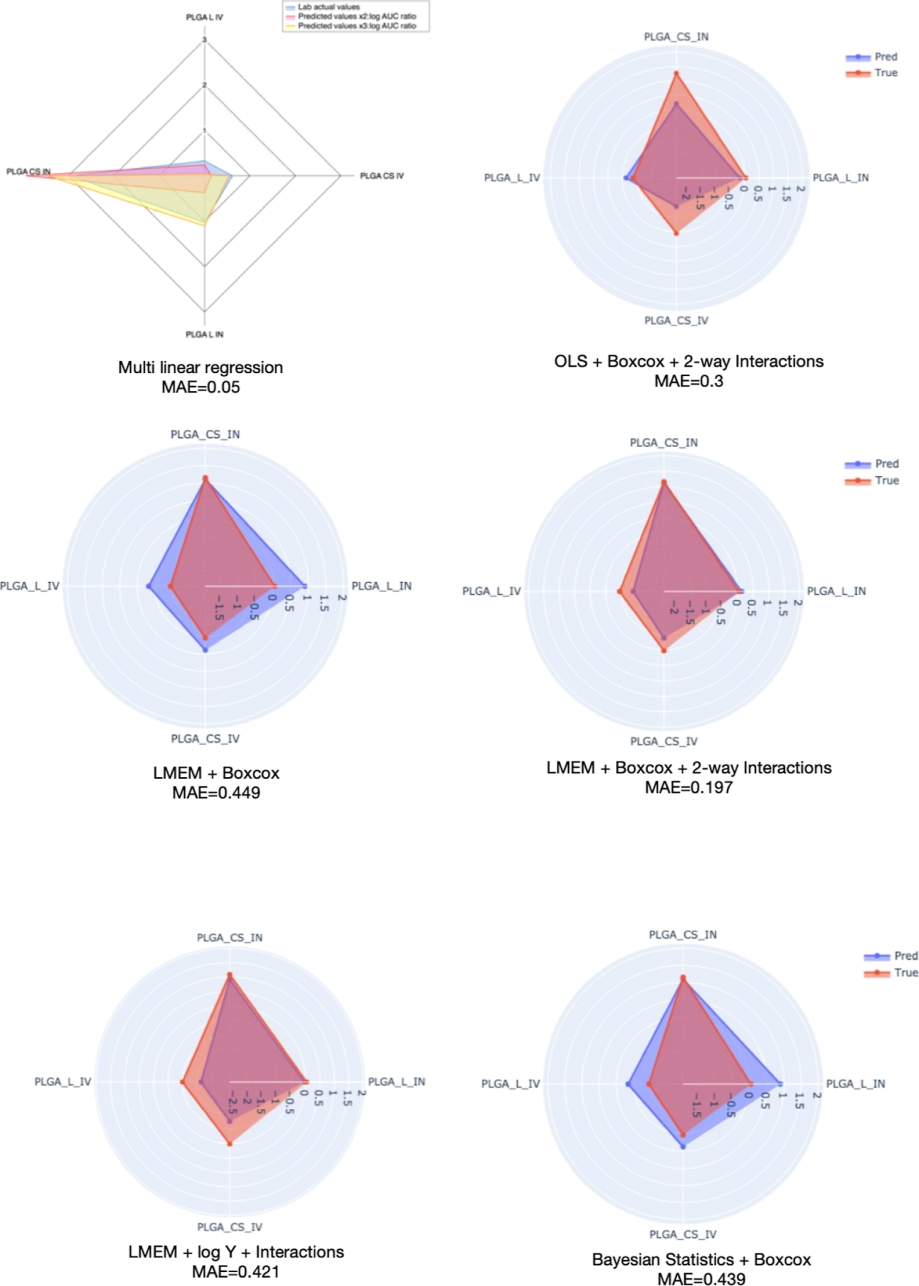
Radar graphs of the predicted AUC values from our top 6 models overladed on top of the true AUC values. The MAE values were added to easily discern the differences between them in terms of superiority.

The results showed that PLGA CS-NPs administered via IN had a significantly higher brain drug uptake index (*Y*) of 2.956 compared with PLGA L-NPs with a *Y* of 1.028. Furthermore, both IN formulations exhibited higher *Y* values compared to the IV administration (1.028 and 2.956 vs 0.329 and 0.614, respectively). This indicates that chitosan, which acts as a mucoadhesive and penetration enhancer due to its positive charge interacting with the negatively charged cell membrane, demonstrated a better brain targeting efficiency. These findings are consistent with previous studies highlighting the effectiveness of the IN route in overcoming the BBB limitations, reducing peripheral side effects, and enhancing therapeutic efficacy.^10,31,32^ The performance of the models was assessed by comparing the observed values to the predictions shown in Table 9 and Figure 5. The LMEM achieved the best overall performance with a validation mean absolute error (MAE) of 0.197. The inclusion of 2-way interactions had a significant positive impact on model performance. Additionally, the Boxcox transformation outperformed the log transformation. Although the GLM showed better performance in training, one of the OLS models demonstrated superior performance compared to all the GLM models. The Bayesian approach also showed comparable performance to the other models.

**Table 9:** True vs Predicted Values for Each of the Prepared Validation NPs from Each of the Fitted Models of Our Statistical Analyses^a^

	true				predicted				
tested model	PLGA L IN	PLGA CS IN	PLGA L IV	PLGA CS IV	PLGA L IN	PLGA CS IN	PLGA L IV	PLGA CS IV	MAE
multivariate linear + *X*^3^ – *X* transformations	1.104	3.46	0.043	0.54	1.028	2.956	0.329	0.614	0.05
OLS + Boxcox + 2-way interactions	0.02	1.26	–0.87	–0.45	–0.18	0.20	–0.63	–1.39	0.3
OLS + 2-way interactions	1.03	2.96	0.365	0.614	1.17	1.25	0.37	–0.342	0.6
OLS + log *Y* + 2-way Interactions	0.029	1.085	–1.007	–0.487	1.17	1.254	0.373	–0.342	0.7
OLS + log *Y* + 2-way Interactions – *X* transformations	0.029	1.085	–1.007	–0.487	1.041	–5.236	0.329	–16.46	4.9
GLM (Tweedie)	1.03	2.96	0.365	0.614	–0.606	1.118	0.798	0.781	0.719
GLM (γ)	1.03	2.96	0.365	0.614	0.500	1.450	0.914	–0.960	0.766
GLM (Gaussian + log link)	1.03	2.96	0.365	0.614	0.953	0.873	0.335	0.576	0.558
LMEM	1.03	2.96	0.365	0.614	4.377	3.626	2.444	1.693	1.792
LMEM + Boxcox	0.029	1.152	–0.954	–0.474	0.892	1.106	–0.331	–0.117	0.449
LMEM + Boxcox +2-way interactions	0.029	1.152	–0.954	–0.474	0.118	1.120	–1.392	–0.883	0.197
LMEM + log *Y* + 2-way interactions	0.029	1.152	–0.954	–0.474	–0.05	0.908	–1.654	–1.271	0.421
Bayesian statistics + Boxcox	0.029	1.152	–0.954	–0.474	0.889	1.095	–0.347	–0.125	0.439
Bayesian statistics + Boxcox + by subject random intercept	0.029	1.152	–0.954	–0.474	–0.759	–0.867	–1.526	–1.056	0.99

a Along with the calculated MAE values.

Figure 3D illustrates the importance of features according to the LMEM model. Factors such as release rate and molecular weight had a negative impact on brain targeting.^33^ Drug solubility and log *P* were found to be influential in predicting BBB permeation according to Norinder,^34^ Haeberlein,^35^ Clark,^36^ and Wermeling et al.^37^ The model also suggests a slightly positive impact on brain targeting when the drug is a P-glycoprotein (P-gp) substrate. Furthermore, controlled release patterns of drugs from NPs were found to have a positive effect on drug accumulation in brain tissue. These findings emphasize the importance of considering drug solubility, P-gp substrate status, and controlled release patterns in NP formulations to enhance brain drug delivery and overcome BBB limitations.^38,39^ Additionally, the inclusion of surfactants and solubility enhancers, such as poloxamers, PVP, and phospholipids (referred to as component (i)), has a significant positive effect on brain targeting, as shown in Figure 3Di. Another factor affecting NP performance is the coating agent (referred to as component (ii)), as illustrated in Figure 3Dii.

Previous studies have highlighted the need for additional solubilization strategies when delivering poorly soluble drugs via the nasal–brain route, where penetration of the nasal mucosa is limited.^40−42^ For example, viscosity-enhancing agents like methylcellulose and sodium alginate can enhance drug retention in the nasal cavity by slowing down the movement of mucus.^43^ Furthermore, incorporating mucoadhesive agents in formulations can reduce the drug passage from the nasal cavity to the pharynx, increasing drug retention. Our data analysis revealed the positive effects of chitosan as a mucoadhesive,^44^ cholesterol as a penetration enhancer,^45^ and sodium alginate as a viscosity enhancer on drug accumulation in the brain.^46^ Additionally, surface coating with PEG polymers can create stealth particles that avoid clearance of NP systems from the bloodstream.^47,48^

Our study did not identify an optimal particle size for nasal–brain drug delivery. However, the physical size of the formulation is likely to play a crucial role in this process. The nasal mucosa exhibits a relatively high degree of permeation flexibility, but there may be a size limit that restricts the passage of particles through factors such as the diameter of olfactory nerve neurons and the primary mechanism of entry through the cell membrane. In contrast, when NPs are administered intravenously, they cannot freely diffuse through the BBB. Instead, they require receptor-mediated transport across the brain capillary endothelium to deliver their contents to the brain parenchyma. It is also important to consider the stability of NPs in biological fluids. These findings are further supported by the positive effect of IN administration on drug accumulation in the brain, as illustrated in Figure 3Diii.

## 4 Conclusions

In conclusion, the development of predictive models for the brain targeting of NPs in CNS clinical disorders is of great importance. In this study, we addressed this challenge by leveraging previous research and assembling a data set of NP formation behavior investigations. Through data mining and analysis, we selected 12 key features related to drug properties, nanocarrier preparation, and nanocarrier properties that were found to significantly impact brain targeting. Various linear regression models, including multiple, generalized, and mixed effect linear regressions, were fitted to the data to predict brain targeting. The models were evaluated using metrics such as *R*^2^ and MAE, and their accuracy was validated against experimental data obtained from in vivo testing of polymeric NPs. The proposed models demonstrated a high accuracy in predicting the brain targeting response. However, there is room for further improvement. Future research should explore more comprehensive tools for data-driven equation extraction, such as SISSO, subgroup discovery, and investigate the applicability domains of the models. Additionally, nonlinear models with nontrivial compositions should be explored to provide a more comprehensive understanding of the underlying mechanisms. By computing the outcome prior to laboratory experiments, our models offer a cost-efficient approach to guide the design of nanocarriers and anticipate their behavior in complex biological environments. This approach helps avoid unintended biological outcomes during clinical applications. However, further advancements in modeling techniques and the availability of a more comprehensive data set will contribute to enhancing the accuracy and applicability of these predictive models.

## Data Availability

The code for the analyses is available at https://github.com/Introvertuoso/BrainTargeting.

## Supporting Information Available

Data showing the relationships between DTP %, DTE %, and other studied features; loading vectors’ data showing that molecular weight and solubility exhibit similar patterns for both administration routes, while the other features display inverted effects; data showing that PC scores and biplot analysis identified outliers within the data set, suggesting their exclusion from further analysis; eigenvalues and proportion of variance distribution data showing a relatively even spread across the predictors, indicating that variability in the data is not concentrated in specific components; and correlation analysis data showing weak correlations between the principal components and response variables, indicating limited influence of the PCs on the responses (PDF)
Physical property data (XLSX)
Physical property data (XLSX)
