# Supplementary material for: A Comprehensive Study on Nanoparticle Drug Delivery to the Brain: Application of Machine Learning Techniques
Source: Mol Pharm. 2023 Dec 7;21(1):333–45. doi: 10.1021/acs.molpharmaceut.3c00880 (PMC10762658; doi:10.1021/acs.molpharmaceut.3c00880)
Supplement: Supplementary file 1 — mp3c00880_si_001.pdf [file mp3c00880_si_001.pdf]

A Comprehensive Study on Nanoparticle Drug Delivery to the Brain: Application of Machine Learning Techniques

Amal Yousfan[a, b], Mhd Jawad Al Rahwanji[c], Abdulsamie Hanano[d], Hisham Al-Obaidi[a]*

[a]The School of Pharmacy, University of Reading, Reading, RG6 6AD, UK

[b]Department of Pharmaceutics and Pharmaceutical Technology, Pharmacy College, Al Andalus University for Medical Sciences, Tartus - AL Kadmous, Syria

[c]Department of Computer Science, Saarland University, 66123 Saarbrücken, Saarbrücken, Germany

[d]Department of Molecular Biology and Biotechnology, Atomic Energy Commission of Syria (AECS), P.O.b. 6091 Damascus, Syria

*E-mail: h.al-obaidi@reading.ac.uk; Tel: +441183786261

Keywords: *nanoparticles; intranasal drug delivery; brain; AUC; prediction, linear regression;* linear mixed-effects

Supporting Information for Publication

1. Methods

1.1 DTP% and DTE%

The calculation of DTP% and DTE% was conducted in this study. DTP% represents the proportion of the drug that directly reaches the brain via the nasal route, specifically through the olfactory or trigeminal nerves, compared to the total amount of the drug that reaches the brain through both direct delivery and systemic administration crossing the blood-brain barrier (BBB). DTP% can range from negative values to a maximum of 100%. Negative values indicate that the drug is more efficiently delivered systemically compared to direct administration via the intranasal route. On the other hand, DTE% specifically compares the direct delivery to the brain. DTE% values above 100% indicate particularly high brain permeability following intranasal administration

Due to the absence of DTE% values for all drugs in certain studies, the following equations demonstrate the calculation of DTE% and DTP% based on the AUC ratio of the brain and blood:

$$DTE\% = \frac{(AUC_{brain}/AUC_{plasma})_{IN}}{(AUC_{brain}/AUC_{plasma})_{IV}} \qquad DTP\% = \frac{B_{in} - B_x}{B_{in}} * 100 \qquad B_x = (B_{iv}/P_{iv}) * P_{in}$$

These equations allow for the estimation of DTE% and DTP% by utilizing the respective AUC values for drug levels in the brain and plasma. The AUC_{Brain} represents the area under the curve for drug concentration in the brain, while AUCPlasma represents the area under the curve for drug concentration in the plasma. B_x corresponds to the fraction of brain AUC contributed by systemic circulation through the blood-brain barrier (BBB) following intranasal administration. By applying these equations, the DTE% and DTP% values can be determined for a comprehensive evaluation of drug delivery mechanisms.

1.2. Exploratory Data Analysis

In order to gain insights into the distribution of variance among the predictors, Principal Component Analysis (PCA) was employed. PCA is a statistical technique that transforms a set of correlated variables into a smaller set of uncorrelated variables called principal components. By analyzing the principal components, we can understand the relative contributions and patterns of variation within the dataset. In this study, PCA was utilized as a tool to examine the distribution of variance across the predictors and identify any underlying patterns or relationships among them.

2. Results and discussion

2.1. The analysis of the correlation between DTE%, DTP% and the studied features

Linear regression analysis was conducted to assess the correlation between DTP% and DTE% with the studied features. The data revealed that the highest R2 values were associated with log P (0.35 for DTP% and 0.4 for DTE%) and molecular weight (0.25 for DTP% and 0.26 for DTE%). Additionally, the results indicated that DTP% and DTE% decrease as particle size and the solubility of the loaded drug increase, with R2 values of 0.31 and 0.37 for particle size, and 0.4 and 0.19 for solubility, respectively.

SI. 1, which depicts the scatter matrix analysis based on a 95% confidence level, shows a positive correlation between DTP% and log P. This observation aligns with expectations, as hydrophobic drugs are generally more permeable across the blood-brain barrier (BBB). Several studies have also examined the impact of log P on DTP% and DTE% and reported a positive correlation between log P and brain targeting (1-3).

2.2. PCA

SI.2 displays Loading plots for both intranasal (IN) and intravenous (IV) administrations, and interestingly, they exhibit a similar pattern with the exception of being vertically fl ipped. Solubility and molecular weight show consistent behavior in both plots. Additionally, log P, solubility, and molecular weight contribute to the variation in PC1, while release, size, zeta potential, and drug carrier contribute to the variation in PC2.

The presence of outliers is evident from the Bipolt and PC scores plot. These outliers were excluded in the subsequent analysis to ensure accurate results. It is worth noting that no individual PC showed a strong correlation with any of the response variables. Furthermore, the proportion of variance after IV and IN administration was not concentrated in the first principal components, indicating that multiple factors contribute to the overall variance in the data.

	Molecular weight	logP	Solubility mg/ ml	Size (nm)	Zeta potential	Release ratio	DTE%	DTP%
**Molecular weight**	1.00	0.26	-0.34		0.13	-0.29	0.25	0.28
**logP Solubility mg/ ml**	0.26	1.00	-0.44	0.06	0.21	-0.22	-0.39	-0.06
**Size (nm) Zeta potential Release ratio**	-0.34	-0.44	1.00	0.05	0.29	0.11	0.01	-0.25
**DTE% DTP%**		0.06	0.05	1.00	0.01	-0.13	-0.10	-0.05
	0.13	0.21	0.29	0.01	1.00	-0.30	-0.20	-0.12
	-0.29	-0.22	0.11	-0.13	-0.30	1.00	0.11	0.05
	0.25	-0.39	0.01	-0.10	-0.20	0.11	1.00	0.65
	0.28	-0.06	-0.25	-0.05	-0.12	0.05	0.65	1.00

SI. 1 Correlation matrix illustrating the relationships between DTP%, DTE%, and the other studied features using Pearson's R correlation coefficient. The analysis reveals a positive correlation between DTE% and logP, indicating that DTE% increases as logP values rise. The correlation matrix provides valuable insights into the interconnected nature of DTP%, DTE%, and the other studied features, shedding light on their relationships and potential implications.

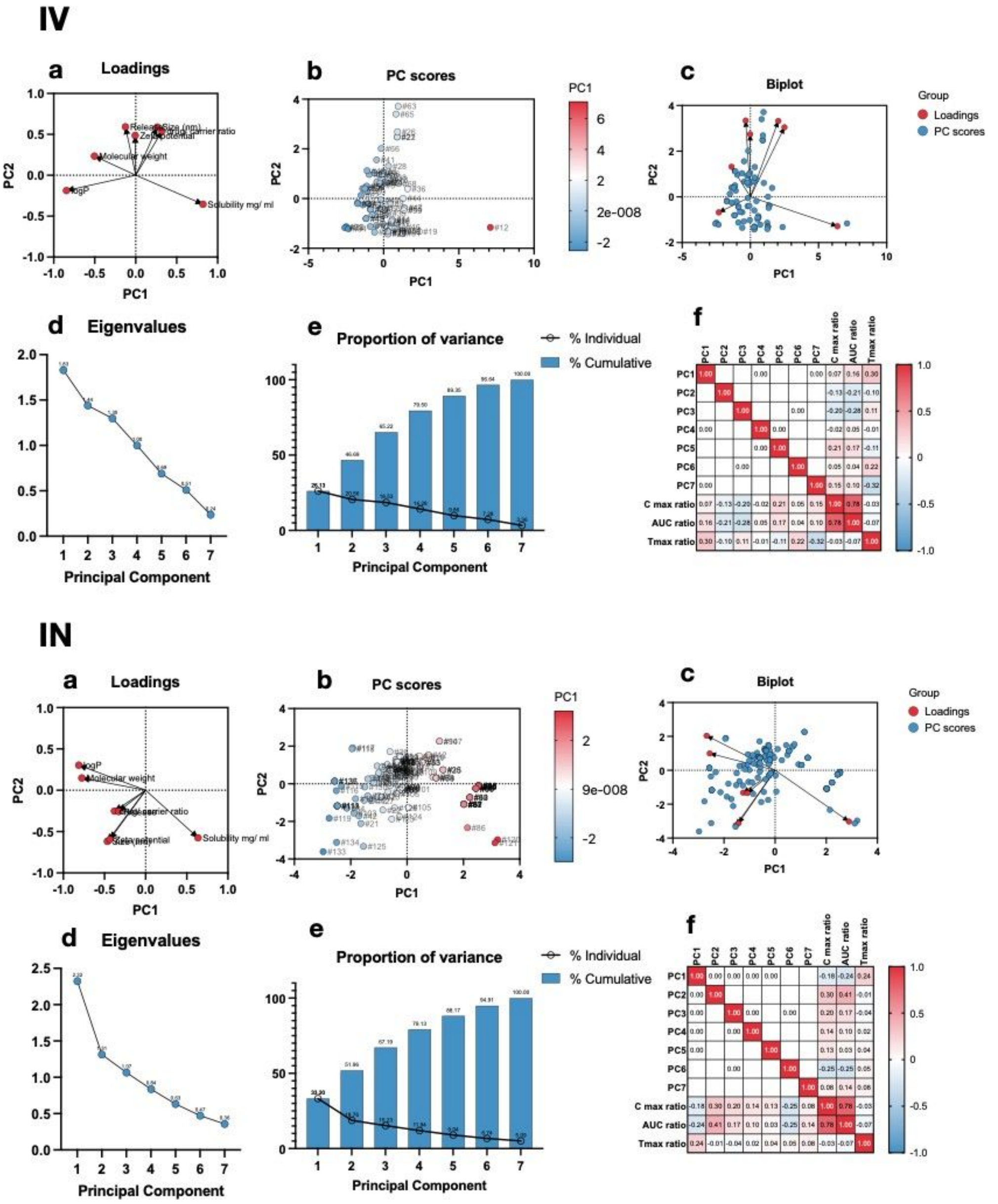

SI. 2 (a) The loading vectors reveal that molecular weight and solubility exhibit similar patterns between the two administration routes, while the remaining features display inverted effects. **(b)** and **(c)** The PC scores and bipolt analysis identify outliers within the dataset, suggesting their exclusion from further analysis to ensure robust results.**(d)** and **(e)** The eigenvalues and proportion of variance distribution show a relatively even spread across the predictors, indicating that the variability in the data is not concentrated in specific components. **(f)** The correlation analysis demonstrates weak correlations between the principal components (PCs) and the response variables, indicating that the PCs have limited influence on the responses.This caption summarizes the key findings related to the relationships, outliers, variance distribution, and correlations observed within the studied features and principal components.

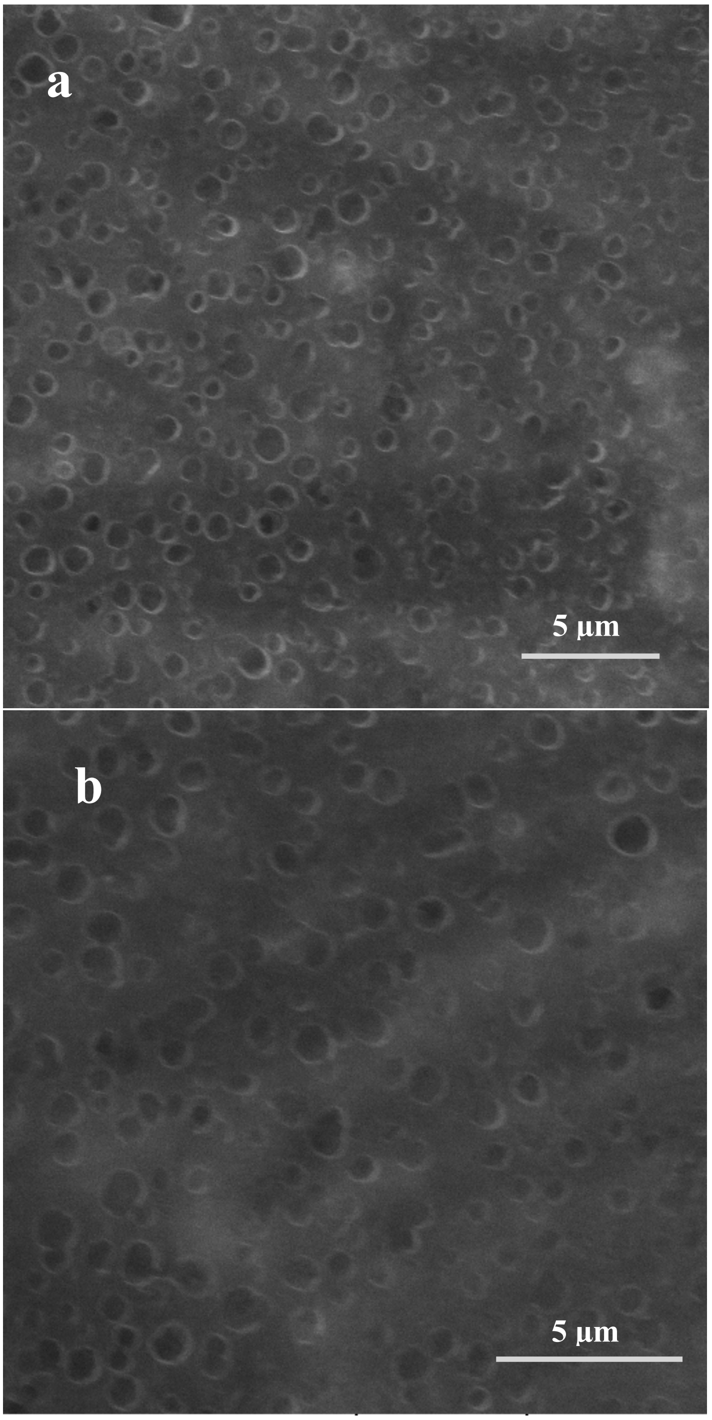

SI. 3 PLGA CS-NPs (a) and PLGA L-NPs (b) tissue observed under scanning electron microscopy.

Bibliography

1. Walton NY, Uthman BM, Yafi K, Kim JM, Treiman DM. Phenytoin Penetration into Brain After Administration of Phenytoin or Fosphenytoin. Epilepsia. 1999;40(2):153-6.

2. Czapp M, Bankstahl JP, Zibell G, Potschka H. Brain penetration and anticonvulsant efficacy of intranasal phenobarbital in rats. Epilepsia. 2008;49(7):1142-50.

3. Marchi N, Betto G, Fazio V, Fan Q, Ghosh C, Machado A, et al. Blood-brain barrier damage and brain penetration of antiepileptic drugs: Role of serum proteins and brain edema. Epilepsia. 2009;50(4):664-77.

4. Feng Y, He H, Li F, Lu Y, Qi J, Wu W. An update on the role of nanovehicles in nose-to- brain drug delivery. Drug Discovery Today. 2018;23(5):1079-88.
